# Endoplasmic reticulum and Golgi stress signaling-mediated regulation of protein secretion

**DOI:** 10.1186/s11658-026-00932-w

**Published:** 2026-05-07

**Authors:** Ketsia Bakambamba, Manon Nivet, Sophie Martin, Elodie Lafont, Eric Chevet, Tony Avril

**Affiliations:** 1 UMR1242 Oncogenesis Stress Signaling, Proteostasis and Cancer Team, INSERM, University of Rennes, 35042 Rennes, France; 2 Centre de Lutte Contre Le Cancer Eugène Marquis, Rennes, France

**Keywords:** Secretory pathway, Protein secretion machinery, Endoplasmic reticulum stress response, Golgi stress response

## Abstract

The eukaryotic secretory pathway (SP) is essential to ensure cellular functions and multicellular communication. The early SP is constituted mostly of the endoplasmic reticulum (ER), the ER–Golgi intermediate compartment (ERGIC), and the Golgi apparatus. These intracellular organelles achieve proper folding and modification of newly synthesized transmembrane and secretory proteins, en route to their final destination, e.g., plasma membrane, endosomes, lysosomes, and the extracellular space. They also integrate quality control systems to ensure export of productively folded proteins and to trigger dysfunctional proteins to degradation. The ER as the first SP compartment is subjected to a precise control of its own homeostasis through signaling of the unfolded protein response. In this review, we provide an overview of the early SP and its regulatory mechanisms, focusing on the ER and Golgi stress-dependent signaling. We contextualize this information within physiological and pathological processes, and discuss how ER and Golgi stress responses might coordinate their regulatory effects across the entire SP.

## Cellular organelles and molecular machineries associated with the early secretory pathway

### The endoplasmic reticulum: the first compartment of the secretory pathway

Approximately one-third of mammalian proteins enter the secretory pathway (SP), which starts at the endoplasmic reticulum (ER). These proteins are synthesized, modified, and matured before reaching their final destination through vesicular trafficking [1].

#### ER protein quality control

**Fig. 1:**
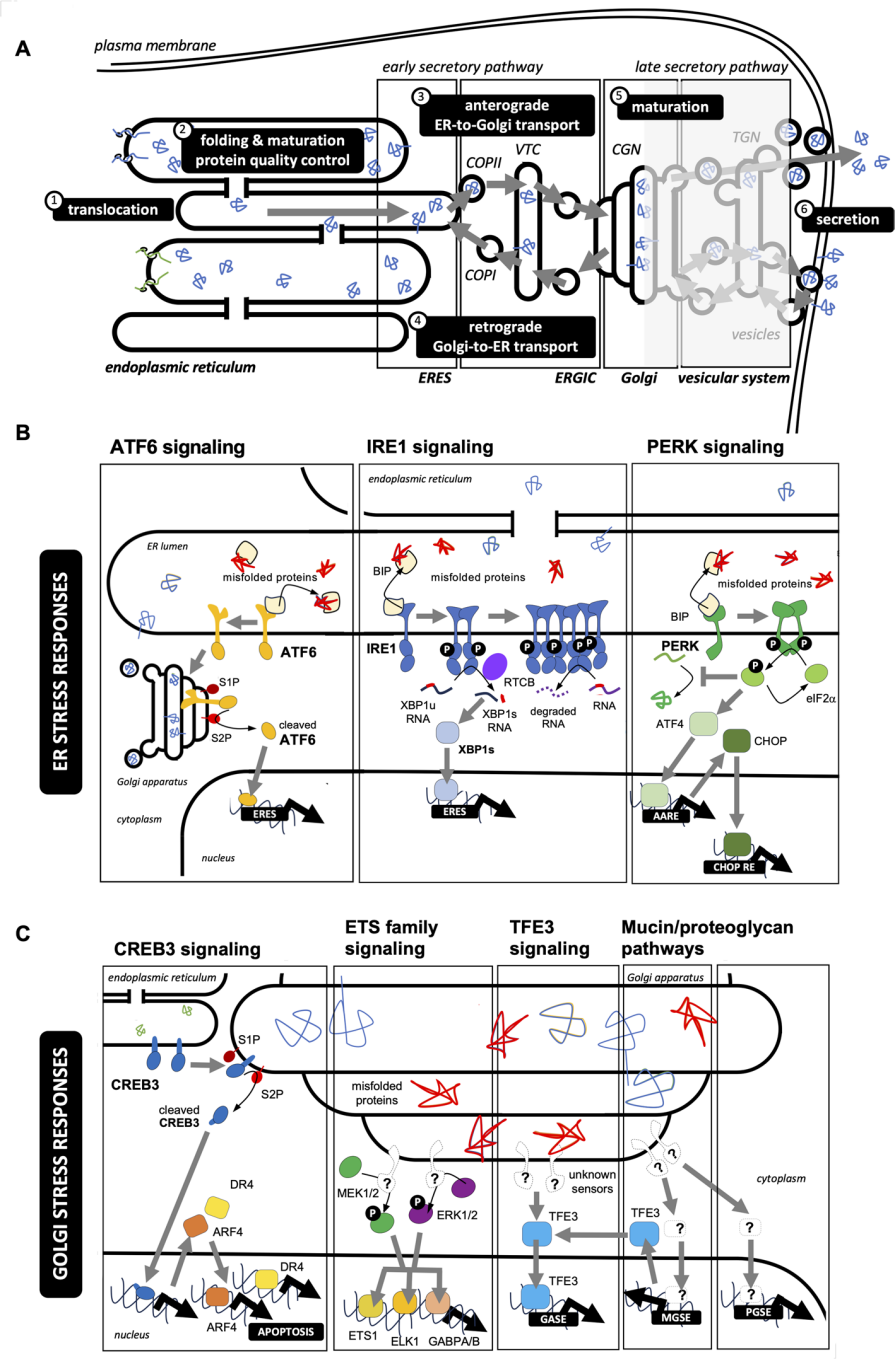
**A** Cellular compartments involved in the early protein SP—Newly synthesized proteins are translocated into the ER membrane (1), where they undergo maturation (2); and are exported from the ER exit site (ERES) to the ER–Golgi intermediate compartment (ERGIC) (3). ER-resident proteins are retrieved via the retrograde ER-to-Golgi pathway (4). Secreted proteins are transported through the Golgi apparatus (5) and delivered to the plasma membrane via vesicular trafficking (6). **B** Molecules involved in the unfolded protein response—Under homeostatic conditions, the chaperone BiP binds and maintains the UPR sensors ATF6, IRE1, and PERK inactive. ER stress induces BiP dissociation, leading to PERK and IRE1 autophosphorylation and activation; and to ATF6 export to the Golgi, where it is cleaved by S1P/S2P proteases. Activated IRE1 mediates the unconventional splicing of XBP1 mRNA with the RTCB ligase, generating XBP1s. PERK phosphorylates eIF2α, resulting in global translational attenuation and selective induction of ATF4. Prolonged ER stress promotes IRE1-dependent RIDD activity, reducing the protein synthesis load. **C** Molecules involved in the Golgi stress response—By a mechanism that remains to be identified, Golgi stress activates the MAPK MEK1/2 and ERK1/2, inducing ETS-family transcription factors; as well as TFE3 and additional pathways, including mucin and proteoglycan signaling. Golgi stress also promotes ER-to-Golgi trafficking of transmembrane protein CREB3, where it is cleaved by S1P/S2P proteases to release its active form

In eukaryotic cells, the early SP initiates at the rough endoplasmic reticulum (ER), where nascent proteins synthesized by ER-bound ribosomes are translocated into the ER lumen through the translocon complexes (Fig. 1A(1)). The ER is the primary site for secretory or transmembrane protein biogenesis, including posttranslational modifications such as *N*-glycosylation and disulfide bond formation, which are essential for correct folding and function [2]. These processes are tightly regulated by ER chaperones and foldases, including thiol-disulfide oxidoreductases and protein disulfide isomerases (PDIAs). Together with the degradation factors, these components constitute the ER quality control (ERQC) system [3] (Fig. 1A(2)). ER-to-cytosol signaling (ERCYS) has recently been described as a novel quality control pathway that refluxes proteins, without degrading them, from the ER to the cytosol, where they can acquire new functions [4, 5]. This process involves ER membrane co-chaperones DNAJB12 and DNAJB14 together with the cytosolic chaperones HSC70 and SGTA to extract proteins from the ER and to release them in the cytosol under a folded conformation [6].

ER chaperones and foldases—Upon entry into the ER, nascent polypeptides are assisted by ER chaperones that promote early folding and maturation [3]. Major ER chaperones include BiP (HSP70 family), GRP94 (HSP90 family), and GRP170 (HSP110 family), all of which contain ER retention motifs, such as KDEL [7], and function in an adenosine triphosphate (ATP)-dependent manner [8, 9]. Their activity is regulated by co-chaperones of the HSP40 family, including DNAJB9, DNAJC3, and DNAJC10. For instance, DNAJC3 enhances BiP ATPase activity [10] and interaction with unfolded substrates [11]. In addition, BiP interaction with DNAJB9 facilitates its binding to the ER stress sensor IRE1 and limits IRE1 activation [12] (see part II.1). Lectin-like chaperones such as calnexin (CANX), calreticulin (CALR), or calmegin (CLGN), together with PDIA3, support the productive folding and quality control of *N*-linked glycoproteins [13–15].

ER degradation machinery—Proteins that fail to fold correctly are eliminated through the ER-associated degradation (ERAD), which involve retrotranslocation to the cytosol and proteasomal degradation [16]. ERAD relies on BiP, lectin chaperones (CANX, CALR, CLGN, EDEM1-3, and MAN1B1), protein disulfide isomerases (P4H1, TXNDC5/11), the co-factors DNAJB9, DNAJC3, and DNAJC10, as well as the E3 ligase HRD1 [17, 18], as reviewed in Refs. [19, 20]. Recent structural insights into the ERAD core machinery has revealed that SEL1L stabilizes HRD1 for substrate ubiquitination, that OS9/XTP3B facilitates glycoprotein recognition, and that DERLIN family proteins contribute to dramatic conformation changes of the HDR1/SEL1L/OS9 complex to facilitate substrate retrotranslocation from the ER to the cytosol for proteasomal degradation [21]. In addition, HDR1 works with the membrane-anchored E2 ubiquitin ligases UBE2J1/2, which sense lipid packing in the ER membranes and consequently ubiquitinate substrates, once they reach the cytosolic face of the ER, revealing a novel cross-talk between protein degradation and lipid signals [22]. Once targeted to the HDR1/SEL1L/OS9 complex, ubiquitinated substrates are terminally extracted by the vasolin-containing protein (VCP) in complex with the ubiquitin recognition factor in ERAD 1 (UFD1) and the nuclear protein localization protein 4 (NPL4), and are then addressed to the proteasome for degradation [23]. The unconventional secretion protein (UPS) pathway also contributes the clearance of misfolded proteins when ERAD is saturated or impaired [24–27]. Larger misfolded proteins that cannot be handled by ERAD are directed to the ER-phagy [28] or ER-to-lysosome-associated degradation (ERLAD) [29] pathways, which rely on autophagy-related machinery, ER-phagy receptors such as FAM134B and RTNL3 [30, 31], and vesicular transport systems directly delivering cargoes to the lysosomes [29].

#### ER exit sites in anterograde protein transport mediated by COPII

Protein export from the ER occurs at specialized smooth ER regions called ER exit sites (ERES) [1, 2, 32–34] (Fig. 1A(3)). At these sites, COPII (coatomer protein II) complexes drive vesicle budding, allowing cargo transport to the ER–Golgi intermediate compartment (ERGIC) [35–37]. The spatial organization of ERES is dynamically regulated by signaling pathways linked to ER stress, calcium, and mechanical cues, affecting ERES components such as SAR1 and SEC16A (reviewed in Ref. [38]). In addition to vesicle formation, COPII components assemble into collars at the necks of ER tubules, facilitating transport of large cargoes such as collagen directly to the ERGIC [39–42]. This process leads to the formation of tunnel-like structures between the ER and the ERGIC [43, 44], involving TANGO1 binding to COPII at the ERES, as well as interactions with the NRZ tethering complex NBAS/RINT1/ZW10 and SNARE proteins, to facilitate the passage of bulky and large cargoes through these tunnels [45].

#### Regulation of secretory proteins exit from the ER

Newly synthesized proteins are either exported from the ER or retained and targeted for degradation if terminally misfolded through the ERAD, ERLAD, or ER-phagy [32, 46]. At the ERES, cargo selection occurs via receptor-mediated transport or the bulk flow, as reviewed in Refs. [47, 48] (Fig. 1A (3)). Cargo receptors such as the mannose-binding lectin ERGIC-53 are transmembrane proteins that link folded cargo to COPII coats and release them in the ERGIC [49–53]. Accessory ER-resident proteins involved in folding can also assist cargo incorporation into COPII vesicles [32, 49, 52, 54]. In contrast, bulk flow allows properly folded proteins lacking retention signals to enter COPII vesicles passively [33, 55, 56], as observed with secreted proteins such as amylase, chymotrypsinogen [57], and albumin [58].

### The ERGIC: a sorting platform between the ER and the Golgi apparatus

Loaded COPII vesicles fuse with each other or with preexisting ERGIC structures [59, 60], forming vesicular tubular clusters (VTCs) that traffic along microtubules toward the *cis*-Golgi. The ERGIC serves as a key sorting hub and the origin of retrograde transport to the ER [2, 61] (Fig. 1A(4)). It contains proteins such as ERGIC-53 members of the p58 and p24 families, which participate in cargo selection and structural organization. ERGIC-53 and p58 may also function as cargo receptors at the ERES. As for the formation of ERSE, COPII vesicle disassembly and fusion with the ERGIC membranes are tightly regulated to ensure efficient cargo trafficking. Importantly, the Trk-fused gene TGF controls the COPII coat dissociation by interacting with the SEC23 subunit [62]. More recently, TUG (for tether containing a UBX domain for GLUT4) has emerged as an important regulator of ERGIC architecture, as its degradation disrupts ERGIC-dependent processes, including autophagy and collagen secretion [63].

COPI-mediated retrograde transport—Retrograde trafficking from the ERGIC to the ER is mediated by COPI coats assembled following small GTPase ARF activation [64, 65]. This pathway enables retrieval of immature and mislocalized proteins as part of the ER/Golgi quality control. Cargo selection depends on retrieval signals, i.e., KKxx or KxKx motifs at the protein C-termini, recognized by COPI components, predominantly the γ-COP subunit [1, 2, 47]. Proteins involved in this process include KDEL receptor, itself containing a di-lysine retrieval motif [2], VIP36, and CALR [66, 67].

### The Golgi apparatus: the final platform of the early SP

The Golgi apparatus is the major site for final protein maturation, including glycosylation, phosphorylation, proteolysis processing, and complex assembly [68]. It also function as a central sorting platform for ER-derived proteins [1, 69] (Fig. 1A(5)).

Protein QC in the Golgi—The Golgi implements its own QC mechanisms to redirect misfolded or unassembled proteins either back to the ER or toward Golgi-associated degradation pathways [68, 70]. COPI-mediated retrieval enables the return of defective proteins [70, 71] such as unassembled immunoglobulins M [72] or major histocompatibility complex (MHC) class I and II molecules [66]. This QC process is pH- and zinc-dependent [73] and involves proteins such as ERP44, which localizes to ERGIC and *cis*-Golgi [71, 72]. RER1 (for retention in ER sorting receptor 1) recognizes transmembrane retrieval signals [74] and participates in COPI-dependent retrograde transport [68, 75, 76].

Golgi-associated degradation—The Golgi also contributes to protein degradation via proteasomal and lysosomal pathways [68]. Membrane proteins can be ubiquitinated by E3 ligases such as NEDD4 and MARCH4 and delivered to lysomes through the endosomal sorting complexes required for transport (ESCRT)-dependent multivesicular bodies (MVB) [68, 77, 78]. Upon Golgi overload condition, luminal proteins such as the Z variant antitrypsin [79] and apolipoprotein B100 [80] can be rerouted to lysosomes by sortilin 1 (SORT1). Defective membrane proteins can also be extracted for proteasomal degradation through endosome and Golgi associated degradation (EGAD) [68, 81, 82], a mechanism dependent on VCP. Although this process has only been described in yeast so far, it might also occur in metazoans since the same molecular actors are present. During Golgi stress, VCP extracts Golgi-resident protein GM130, a tethering Golgi matrix protein, for degradation by the proteasome [68, 77]. Additional factors, including the Golgi-resident α1,2-mannosidase MAN1A [83], DSC2 complexes [81], and ERGIC structural protein TUG (for tether containing UBX domain for GLUT4) [63], further contribute to Golgi QC balancing conventional and unconventional protein secretion via ERGIC-derived autophagosome biogenesis as well as degradative pathways.

## ER and Golgi stress responses

### The ER stress response and its sensors

Cellular conditions such as acute increases of protein synthesis (e.g., during oncogenesis) or nutrient deprivation can overwhelm the ER folding capacity, leading to the accumulation of unfolded or misfolded proteins and the activation of ER stress [84–87]. ER stress affects multiple cellular functions including metabolism, migration, and secretion. To restore ER homeostasis, cells activate the unfolded protein response (UPR), an adaptative signaling program that enhances folding and degradation capacities while reducing protein synthesis [88]. When the ER stress is prolonged or unresolved, the UPR can initiate cell death pathways [88, 89]. By preventing the secretion of aberrant proteins and limiting proteotoxicity, the UPR plays a critical role in maintaining cellular and organismal homeostasis [90]. The UPR is mediated by three ER-resident transmembrane sensors: the activating transcription factor 6 (ATF6), the inositol-requiring enzyme 1α (IRE1 also known as ERN1), and the protein kinase RNA-like ER kinase (PERK, also known as EIF2AK3) [85–87] (Fig. 1B). The mode of activation of the UPR sensors is still currently under debate. Under basal conditions, these sensors are kept inactive through association with the chaperone BiP [87]. During ER stress, unfolded proteins are recruited to BiP by the DNAJB9 co-chaperone molecule, promoting BiP ATPase activation and release of ER stress sensors [91]. In addition, accumulating evidence suggests that misfolded proteins may directly bind and stabilize IRE1 (and possibly PERK) dimers, contributing to their activation (reviewed in Ref. [92]). Sensor activation involves BiP dissociation [93], redox-dependent conformation changes [93] mediated by PDIA5 [94] and/or TXNDC12 [95] for ATF6 [93]; and homodimerization followed by autophosphorylation for IRE1 and PERK. Once activated, each sensor initiates distinct signaling pathways that collectively aim at restoring ER homeostasis [85–87].

ATF6, an ER transmembrane sensor that delocalizes into the Golgi to signal—Upon ER stress, ATF6 undergoes redox-dependent dimerization, facilitated by TXNDC12, and is exported to the Golgi apparatus, where it is sequentially cleaved by the Golgi resident proteases S1P and S2P [85–87, 95] (Fig. 1B). This processing releases the cytoplasmic ATF6f, a potent transcription factor that binds ER stress response elements (ERSE) and induces expression of genes involved in ER homeostasis, protein degradation, and redox regulation [87, 96].

IRE1, a central UPR component with kinase and endoribonuclease activities—IRE1 is the most evolutionarily conserved UPR sensor and plays a central role in ER stress signaling [85–87, 97] (Fig. 1B). IRE1 is a type I ER resident transmembrane protein containing cytosolic serine/threonine kinase and endoribonuclease (RNase) domains, both activated by oligomerization and autophosphorylation [98]. ER stress promotes IRE1 dimerization and, under sustained stress, higher-order clustering, which enhances its phosphorylation and activity [99]. Activated IRE1 catalyzes the unconventional splicing of X-box binding protein 1 (XBP1) mRNA, generating a potent transcription factor XBP1s with the help of the RNA ligase RTCB [100, 101]. XBP1s induces ERSE-containing genes that expand ER folding capacity and promote ER-associated degradation [102]. Under prolonged stress, IRE1 RNase also mediates the regulated IRE1-dependent decay of RNA (RIDD), degrading ER-proximal mRNAs or microRNAs to reduce protein load [103]. For instance, degradation of miR-17 stabilizes TXNIP mRNA, increasing TXNIP expression and promoting terminal programmed cell death [104]. A related pathway, named RIDDLE (for RIDD lacking endomotif), targets RNA lacking canonical XBP1-like cleavage motifs and is controlled by oligomerization state of activated IRE1 [105]. In parallel, IRE1 kinase activity links ER stress to JNK (for c-Jun N-terminal kinase) signaling through interacting with TRAF2 (for TNF receptor-associated factor 2) [106].

PERK, an ER stress sensor that attenuates RNA translation—Upon activation, PERK phosphorylates the α-subunit of the eukaryotic initiation factor 2 alpha eIF2α (Fig. 1B), leading to global attenuation of protein synthesis and reduced ER load [85–87]. Despite this translational repression, selected mRNAs containing upstream open reading frames, including ATF4 (for activating transcription factor 4), remain efficiently translated. ATF4 induces transcription of genes involved in amino acid transport, metabolism, autophagy, redox homeostasis, and oxidative stress adaptation, notably through cooperation with CHOP [85–87]. In addition, PERK directly phosphorylates NRF2, thereby modulating the cellular antioxidant response [107].

### The Golgi stress response and its associated signaling pathways

As described for the ER, excessive protein production and trafficking demands can overwhelmed the Golgi, impairing protein modification and export; and triggering Golgi stress [108, 109]. Although less characterized than the UPR, the Golgi stress response is emerging as an adaptive signaling program that preserves Golgi function under conditions such as glycosylation defects, lipid imbalance, disrupted vesicle transport, or secretory overload [109]. While Golgi stress sensors remain unidentified, several Golgi-associated signaling pathways have been described. These pathways involve kinases and transcription factors that regulate genes controlling Golgi structure, membrane dynamics, vesicle trafficking, and glycosylation [108, 110, 111].

CREB3, an ER-resident protein that mediates Golgi stress-dependent cell death—Upon Golgi stress, the ER-resident transmembrane CREB3 is transported to the Golgi, where it is cleaved by S1P and S2P proteases, similar to ATF6. The released cytosolic fragment translocates to the nucleus and induces expression of Golgi-associated genes such as *ARF4* and *DR4*, promoting Golgi stress-induced cell apoptosis [112] (Fig. 1C).

ETS family-associated signaling in Golgi stress-induced apoptosis—Several ETS family transcription factors, including ELK1, ETS1, and GABPA/B, are activated downstream of the mitogen-activated proteins kinase (MAPK) pathway via MEK1/2 and ERK1/2 during Golgi stress (Fig. 1C) [108, 110, 111]. These factors also participate in spliceosome regulation and promote Golgi stress-dependent apoptosis through alternative splicing of MCL1 [113].

TFE3 signaling regulating Golgi structure and glycosylation—Golgi stress induced by altered pH or sialic acid deprivation activates the transcription factor TFE3 (for TF binding to immunoglobulin heavy constant mu enhancer 3) [114]. TFE3 upregulates genes containing Golgi apparatus stress response element (GASE), including those encoding structural Golgi proteins such as *GOLGA2* and *GOLGB1* (also named *GM130* and *giantin*, respectively) [114], vesicular trafficking components (*RAB20* and *STX13*), and enzymes involved in *N*-glycosylation. TFE3 can also be induced by mucin signaling through the mucin-type Golgi stress response element (MGSE) motif, although the upstream sensors and transcription regulators remain incompletely defined [108, 115] (Fig. 1C).

Golgi stress-dependent regulation of protein glycosylation—Golgi stress activates additional pathways that reinforce protein glycosylation capacity [108, 115]. The proteoglycan- and mucin-specific stress responses induce genes containing PGSE (for proteoglycan-type Golgi stress response element) and MGSE motifs, respectively, glycosyltransferase and sulfotransferase. While these pathways enhance glycosylation efficiency, their upstream sensors and transcription factors remain unidentified [108, 115] (Fig. 1C).

Putative sensors linked to Golgi mechanics and lipid homeostasis—While Golgi stress sensors remain unidentified, increased secretory load and lipid imbalance elevate Golgi membrane tension. This mechanical stress triggers overexpression of GOLPH3 (for Golgi phosphoprotein 3), which binds phosphatidylinositol-4-phosphate (PI4P) and the unconventional myosin MYO18A, coupling the Golgi to the actin cytoskeleton. This interaction promotes Golgi elongation or fragmentation, increased trafficking, and stress tolerance [116]. GOLPH3 is also phosphorylated by the DNA-dependent protein kinase (DNA-PK), further enhancing its interaction with PI4P and MYOA18 [117]. Additionally, the PI4 kinase PI4KIIIβ is recruited at the Golgi via PI4P and ARF1 (for the ADP-ribosylation factor 1), activating effectors involved in lipid transport and vesicle trafficking [118, 119].

## Control of the early SP upon ER and Golgi stress

### ER and Golgi stress-induced remodeling of early SP organelles

Beyond transcriptional reprogramming [85, 86], ER and Golgi stress alter the morphology and function of early SP organelles, including ER, ERGIC, and Golgi apparatus; and promote the formation of stress-associated cellular structures.

ER morphology remodeling during ER stress—ER stress induces expansion of ER cisternae and membranes [120, 121], generating additional ER sheets and tubules [122–125]. The process is regulated by the IRE1/XBP1s branch of the UPR through increased lipid biosynthesis [122], allowing accumulation of newly synthesized chaperones and foldases to alleviate ER stress [122–125]. In renal cells, for example, hyperosmotic stress activates the IRE1/XBP1s–SREBP1/2 axis, promoting lipogenesis via LPIN1/2 and DGAT1; and ER membrane expansion [126]. ER stress also reduces the number of ERES, contributing to the formation of SEC bodies, membrane-less granules that act as a protective reservoir for ERES components in *Drosophila* cells [127, 128]. Upon prolonged ER stress, ER membranes can reorganize into multilamellar ER whorls in a SAR1/SEC22/PERK-dependent manner. Additionally, ER stress in *Saccharomyces cerevisiae* [4] and in mammals [5, 129] can induce protein reflux from the ER to the cytosol, a process dependent on IRE1 and DNAJB12/DNAJB14 proteins, allowing the relocalization of ER proteins such as AGR2 [6].

ER stress-dependent remodeling of ERGIC and Golgi—ER stress profoundly alters ERGIC and Golgi architecture, slowing ER-to-Golgi protein trafficking [130]. Golgi ribbons and cisterna stacks disassemble, and COPII-coated vesicle formation is reduced, as revealed by fewer SEC31 puncta [130]. Disruption of cargo receptors such as ERGIC53 and SURF4 similarly reduces ERGIC abundance and induces Golgi fragmentation [131].

Stress-induced formation of novel cellular structures—Autophagy is a major adaptive response to ER stress, particularly in pathological contexts such as cancer and hypoxia where cellular oxygen availability can be limited. UPR signaling induces expression of key autophagy regulators, including Beclin-1 (via PERK/ATF4 [132]) and ATG5/12 (via CHOP [133] and PERK/ATF4 [134, 135], respectively), promoting autophagosome formation. UPR also triggers selective ER autophagy, mediated by VAP proteins [28], and in yeast, by IRE1-induced EPR1, which links ER membranes with ATG8-positive autophagosomes [28]. In *Drosophila* cells, nutrient deprivation induces accumulation of SEC16A in SEC bodies via SIK1/2, IRE1, and PERK activation, to preserve secretory capacity during stress and enable rapid recovery upon refeeding [128, 136, 137].

Golgi morphology alterations during Golgi stress and consequences on protein secretion—ER or Golgi stress frequently leads to Golgi fragmentation, characterized by disruption of the ribbon structure and uncoupling of the stacks [108]. Depending on the severity and context, this process can be reversible or irreversible. Persistent Golgi fragmentation is associated with pathological conditions, including neurodegenerative diseases, cancer, and inflammation; and often results in defective protein glycosylation. Golgi architecture and function are maintained by Golgi reassembly stacking proteins (GRASPs), notably GRASP65 and GRASP55 [138–140]. GRASP depletion disrupts glycosylation and activates Golgi stress signaling [141]. Paradoxically, loss of GRASP65 prevents cisternal stacking and accelerates protein trafficking but also causes protein missorting, as illustrated by aberrant secretion of cathepsin D and mannose 6-phosphate receptor [141]. Pharmacological agents such as sorafenib similarly impair secretion by inducing Golgi fragmentation through inhibition of p97/VCP phosphorylation in hepatocellular carcinoma cells [142].

### UPR and Golgi stress-dependent modulation of the early SP

**Fig. 2:**
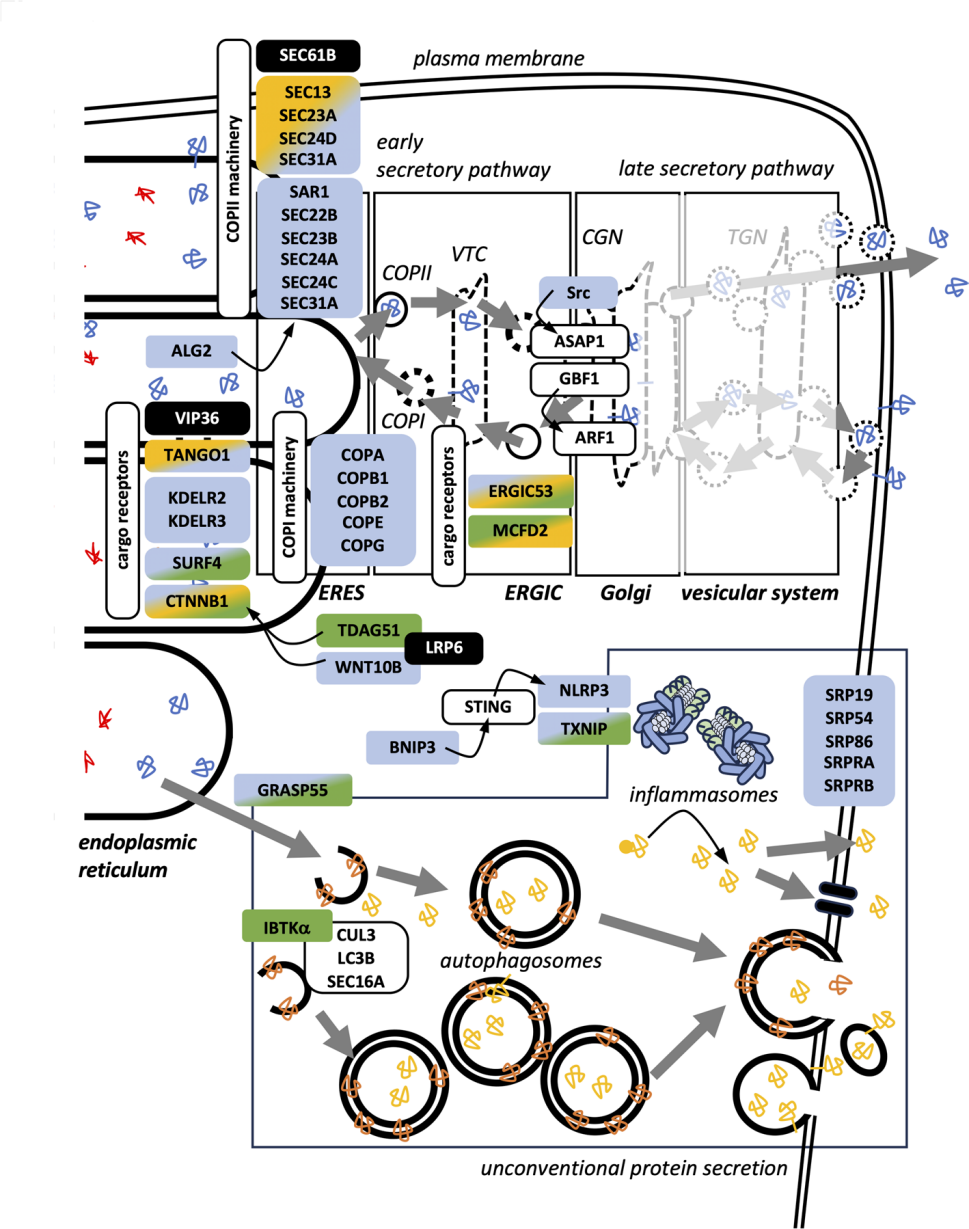
Molecular actors of the early SP regulated by the UPR signaling—Activation of the three ER stress sensors promotes the expression of cargo receptors, components of the COPI and COPII machineries, Golgi-associated proteins, and molecules of the UPS pathway, including inflammasome components. Molecules regulated by ATF6, IRE1, and PERK signaling are highlighted in yellow, blue, and green, respectively

Although the direct regulation of secretory machinery by stress sensors has recently explored, accumulating evidence shows that ATF6, IRE1, and PERK modulate multiple components of the early SP under physiological and pathological contexts such as inflammation, infection, chronic liver diseases and, cancers (Fig. 2). Detailed regulation of protein translocation, quality control, and degradation is reviewed elsewhere [3, 68, 143].

UPR-dependent regulation of COPII machinery at ERES—UPR signaling regulates COPII components at multiple levels. In mammalian cells, XBP1s and ATF6 induce expression of COPII subunits, including SEC13, SEC23A/B, SEC24A/C/D, and SEC31A, as well as regulators such as ALG2 that stabilize COPII assembly at the ERES [32, 144]. Similar regulation occurs across species, including plants and fish, where IRE1/XBP1s homologs or ATF6-like sensors promote COPII-associated proteins (i.e., SEC13, SEC23A/24D, and SEC31A) during stress [145–147]. ATF6 also controls SEC16 splicing via SNRPB, resulting in reduced protein secretion [148]. Notably, UPR exerts opposing effects on secretion pathway by enhancing COPII expression while reducing ERES numbers and expanding ER membranes, thereby limiting export of misfolded proteins.

UPR-dependent regulation of cargo receptors and accessory molecules—Several cargo receptors involved in ER–Golgi transport, including ERGIC-53, MCFD2, VIP36, KDELRs, and SURF4, are regulated by UPR signaling, predominantly by ATF6 and IRE1/XBP1s branches [144, 149–154] (Fig. 2). These receptors facilitate cargo export or ER protein retrieval and are modulated in diverse contexts such as inflammation, heat shock, lactation, and fibrosis [154–156]. Large cargo export factors, including TANGO1 and its accessory molecules, are also induced by UPR signaling to support collagen secretion during stress [43, 145, 157] (Fig. 2).

UPR-dependent regulation of ERGIC–Golgi retrograde trafficking—XBP1s induces expression of multiple COPI subunits involved in retrograde trafficking from the Golgi to the ER [144] (Fig. 2). ER stress also disrupts COPI function through an IRE1–SRC–ASAP1 signaling axis that enhances ARF1 activation [158, 159], but leads to KDELR dispersion and impaired retrograde transport [160, 161]. Together, these mechanisms illustrate how ER stress reshapes bidirectional trafficking to balance proteostasis and secretion.

ER and Golgi stress regulation of unconventional protein secretion—ER and Golgi stress responses can regulate unconventional proteins secretion (UPS) (reviewed in [27, 162]). ER stress induced by misfolded or mutated proteins can activate “Golgi-bypass” pathways that enable direct ER-to-plasma membrane trafficking. Mechanistically, ER stress promotes the expression, phosphorylation, and relocalization of GRASP55 to the ER, where it recognizes PDZ-domain-containing cargoes such as mutant CFRT [163, 164] and facilitates Golgi-independent export. Similarly, ER stress-dependent upregulation of the co-chaperone DNAJC14, acting in concert with HSP70, promotes Golgi bypass of mutant pendrin [165]. Recent evidence implicating the kinase domain of IRE1 in UPS regulation [166], together with PERK- and IRE1-mediated activation of exosome secretion via inhibition of multivesicular body degradation [167], further supports the notion that ER and Golgi stress signaling actively reprograms alternative secretory routes.

### Hypothetical perspectives on the coordinated regulation of the SP machineries by ER and Golgi stress signaling pathways

The UPR is currently the best-characterized adaptive signaling pathway that regulates the early SP, primarily by modulating protein folding capacity, ER-associated degradation, and ER-to-Golgi transport. In contrast, the contribution of the Golgi stress response to SP regulation remains underexplored. Nevertheless, emerging evidence suggests that the ER and Golgi stress responses may act in a coordinated and spatially organized manner to regulate SP at multiple steps in protein secretion.

**Fig. 3:**
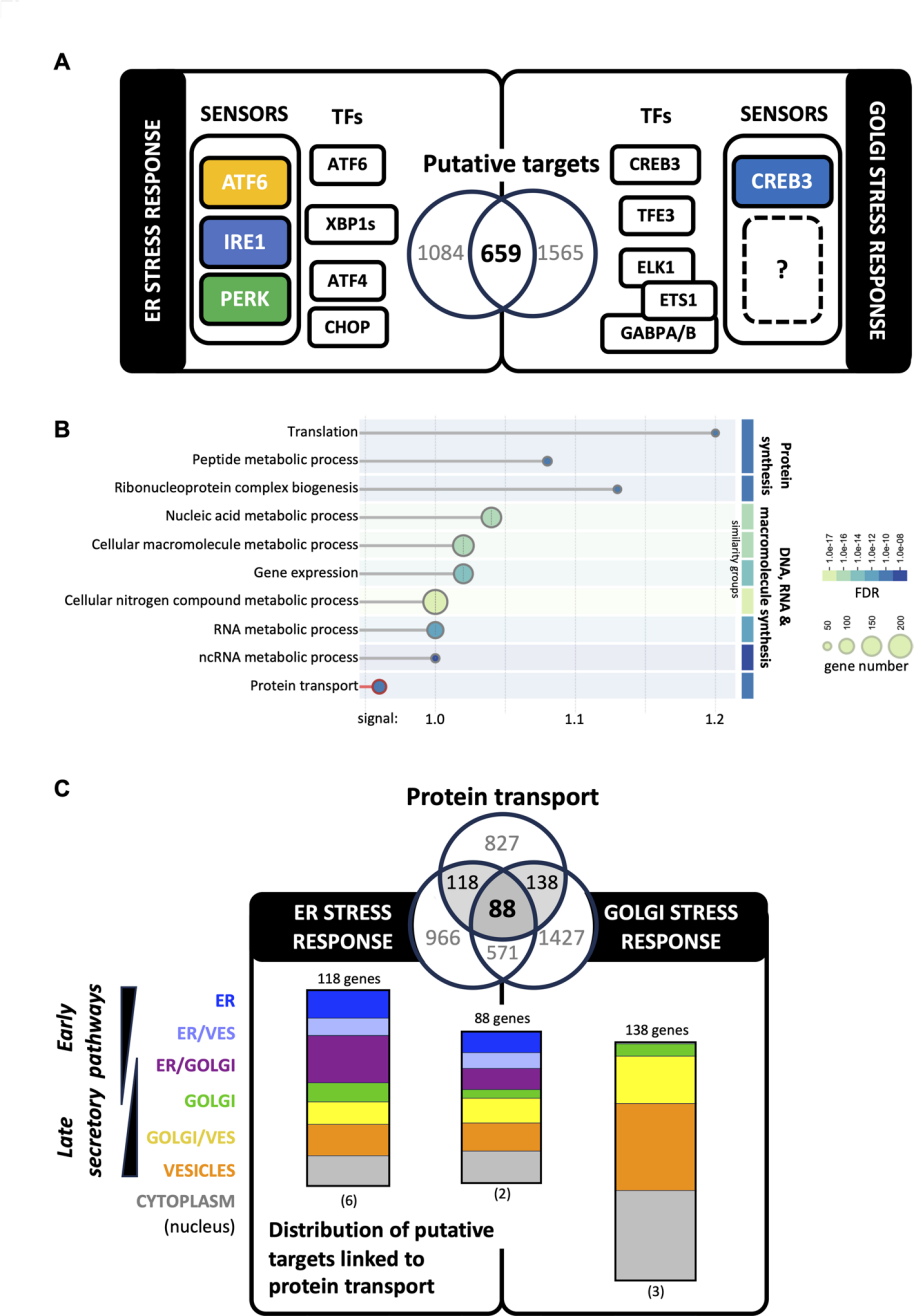
**A** Putative targets of transcription factors induced by ER and Golgi stress signaling—Target genes for transcription factors (TFs) induced by the UPR sensors (i.e., ATF6, XBP1s, and ATF4/CHOP generated following activation of ATF6, IRE1, and PERK) and those of Golgi stress-associated TFs (i.e. ETS1, ELK1, and GABPA/B (from the ETS family), as well as CREB3 and TFE3) were obtained from ChIP-Atlas (https://chip-atlas.org/) and intersected. **B** Functional annotation of putative target genes induced by both ER and Golgi stress signaling—The common set of genes regulated by both ER and Golgi stress responses was analyzed for functional enrichment using the String database (https://string-db.org/), revealing associations with gene expression, translation, nucleic acid and peptide metabolism, protein synthesis, and protein transport. **C** Spatial distribution of putative target genes involved in protein transport and induced by ER and Golgi stress signaling—Target genes of ER and Golgi stress-associated TFs were intersected with genes involved in protein transport (GO term 0015031). Genes specifically and commonly induced by ER and Golgi stress responses were localized using the GeneCards database (https://www.genecards.org)

Do ER and Golgi stress responses lead to similar outputs?—To answer this question, we compiled putative target genes of the transcription factors associated with ER and Golgi stress responses (GSR) using the ChIP-Atlas database. Integrative analyses of publicly available datasets from reveal that 1743 and 2224 genes are putative targets of the transcription factors induced by ER and Golgi stress signaling, respectively (Fig. 3A). As expected, UPR leads to known biological processes associated with misfolding proteins. In contrast, GSR is linked to RNA biology as such RNA processing, splicing, and translation (data not shown). Importantly, both ER and Golgi stress signaling may regulate a common set of genes (*n* = 659) involved in gene expression, translation, nucleic acid and peptide metabolism, protein synthesis, and protein transport (Fig. 3B).

Do ER and Golgi stress signaling converge to regulate protein transport? Among the genes involved in protein transport (1171 genes according to GO term 0015031), 206, 226, and 88 genes are putative UPR targets, GSR targets, and both UPR/GSR targets, respectively (Fig. 3C). Notably, ER and Golgi stress signaling appear to exert spatially distinct control over genes involved in protein transport: the ER stress pathway might influence genes distributed broadly from the ER to the vesicles associated with protein trafficking, whereas the GSR might exclusively regulate genes localized to the Golgi and vesicular system (Fig. 3C). Collectively, these findings support a model in which ER and Golgi stress response cooperatively remodel both conventional and unconventional secretory pathways at distinct spatial levels.

Future directions—Further work will be required to define the molecular hierarchies, signaling crosstalk, and context dependence of these regulatory circuits, and to determine how both ER and Golgi stress signaling pathways impact the early and late SP, as well as the unconventional protein secretion, under physiological and pathological conditions. One could then anticipate proposing pharmacological tools to modulate those mechanisms for therapeutic purposes. While a large arsenal of pharmacological molecules targeting the UPR sensors are already available, identifying Golgi stress sensors and developing dedicated pharmacological agents will be required.

## Conclusions

The regulation of protein secretion by ER and Golgi stress signaling represents a fundamental aspect of cellular homeostasis with broad implications for human disease. Disruptions of proteostasis and secretory pathway function are implicated in cancer as well as metabolic, skeletal, cardiovascular, immunological, and neurodegenerative disorders, underlining the pathological consequences of impaired protein folding and trafficking [34, 90]. Continued investigation into ER–Golgi stress signaling networks will not only advance our understanding of SP biology but may also uncover novel therapeutic and diagnostic opportunities aimed at restoring proteostasis in disease contexts.

## Data Availability

Not applicable.
